# Electrical Impedance Plethysmography Versus Tonometry To Measure the Pulse Wave Velocity in Peripheral Arteries in Young Healthy Volunteers: a Pilot Study

**DOI:** 10.2478/joeb-2021-0020

**Published:** 2021-12-30

**Authors:** A. I. P. Wiegerinck, A. Thomsen, J. Hisdal, H. Kalvøy, C. Tronstad

**Affiliations:** 1 Department of Physiology, Radboud University Medical Center, Nijmegen, Netherlands; 2 Department of Vascular Surgery, Division of Cardiovascular and Pulmonary Diseases, Oslo University Hospital, Oslo, Norway; 3 Institute of Clinical Medicine, Faculty of Medicine, University of Oslo, Oslo, Norway; 4 Department of Physics, University of Oslo, Oslo, Norway; 5 Department of Clinical & Biomedical Engineering, Oslo University Hospital, Oslo, Norway

**Keywords:** electrical impedance plethysmography (IPG), pulse wave velocity (PWV), bioimpedance, tonometry, peripheral arteries

## Abstract

The leading cause of health loss and deaths worldwide are cardiovascular diseases. A predictor of cardiovascular diseases and events is the arterial stiffness. The pulse wave velocity (PWV) can be used to estimate arterial stiffness non-invasively. The tonometer is considered as the gold standard for measuring PWV. This approach requires manual probe fixation above the artery and depends on the skills of the operator. Electrical impedance plethysmography (IPG) is an interesting alternative using skin surface sensing electrodes, that is miniaturizable, cost-effective and allows measurement of deeper arteries. The aim of this pilot study was to explore if IPG can be a suitable technique to measure pulse wave velocity in legs as an alternative for the tonometer technique. The PWV was estimated by differences in the ECG-gated pulse arrival times (PAT) at the *a. femoralis, a. popliteal, a. tibialis dorsalis* and *a. dorsalis pedis* in nine healthy young adults using IPG and the SphygmoCor tonometer as a reference. The estimated PWV results from bioimpedance and the tonometer were fairly in agreement, and the beat-to-beat variability in PAT was similar. This pilot study indicates that the use of IPG may be a good alternative for estimating PWV in the legs.

## Introduction

The leading cause of health loss and deaths worldwide are cardiovascular diseases (1). A predictor of cardiovascular diseases and events is the arterial stiffness, especially in central arteries such as the aorta (2,3). Previous studies have demonstrated that pulse wave velocity (PWV) is a valid marker for arterial stiffness that can be measured non-invasively (4). The PWV is the traveling speed of the pulse wave propagating through the arterial system. A high PWV is associated with a stiffer artery (5). The PWV between the carotid and femoral arteries has been considered as the “gold-standard” measurement of arterial stiffness in the aorta (3). In addition to aortic PWV having clinical value, measurements of peripheral PWV may also have a clinical value. In a study by Lee *et al*. it was demonstrated that in patients with chronic kidney disease that have both a high PWV in the central and peripheral arteries were associated with a rapid decline in kidney function (6). Also studies with diabetes patients show the clinical relevance of PWV in the legs. Diabetes patients have an increased risk of developing peripheral artery disease (PAD). Different studies with diabetes patients with and without any symptoms of PAD have shown to have stiffer femoral and popliteal arteries compared to a healthy control group (2,7, 8, 9).

Moreover, another study with diabetes patients found that peripheral neuropathy is associated with a high brachial-ankle PWV (10). As a high PWV in the legs is associated with different diseases, it is of clinical relevance that the PWV is measured with a good method.

All methods to estimate PWV rely on the measurement of a pulse wave, which is commonly acquired by pressure, distension, or flow waves. Different methods and techniques have been used to measure PWV, i.e. the PWV are the PulsePen® (tonometry and electrocardiogram (ECG)), Complior® (piezoelectric pressure mechanotransducers), SphygmoCor® (tonometry and ECG), Arteriograph® (oscillometric pressure curve analysis), photoplethysmography (infrared probes), ultrasound (Doppler waveforms simultaneously or with ECG), magnetic resonance imagining (MRI, aortic systolic flow wave at two different sites) and invasive measurement (catheterization from peripheral artery with angiography). Among these, pressure sensors such as the tonometer is considered the gold standard for measuring PWV (11). This approach needs holding or mechanical fixation of a probe above the artery during measurement, where operating skills affect the measurement and a trained operator is needed. Typically, these probes are directly placed on the patient’s tissue at two different arteries on a site where the pulsation is easily felt. This gives an average PWV for that segment of the arterial system, but could cause distortion the waveform and might be uncomfortable in some patients (11,12).

Another method is electrical impedance plethysmography (IPG), which is a kind of bioimpedance measurement. Instead of relying on mechanical contact to pick up the pressure waveform, the IPG technique relies on electrical contact through electrodes attached to the skin, using an applied weak electrical current and voltage sensing. The IPG method relies on changes in the ability of the tissue to conduct alternating current and is sensitive to blood volume changes (plethysmography), blood flow and blood composition (haematocrit). Either by sensing the IPG waveform at two arterial sites simultaneously, or together with an electrocardiography (ECG) the PWV can be estimated by measuring the time delay (pulse arrival time (PAT)) and distance between the two sites (13). This could be a promising alternative for the already existing methods, as the IPG is a simple, non-invasive and a low-cost method. The instrumentation and electronics required for IPG measurement is relatively simple, enabling development of a miniaturized device. Another advantage of the IPG is the possibility to observe a pulse in every artery, despite the depth of the artery (14). This could mean that bioimpedance could be used on places where the artery is difficult to detect for other devices.

Aria *et al*. showed in 2019 that using IPG for PWV measurement is a suitable and practical method, but that comparison with other techniques is still needed (14). A suitable comparison is a tonometer, regarded as one of the golden standard for PWV measurement (11). It is suitable as it can be used for every superficial palpable artery (5). The aim of this study was therefore to explore if the bioimpedance IPG can be a suitable technique to measure pulse wave velocity in legs as an alternative for the tonometer technique. The hypothesis is that the IPG is comparable to tonometry for the measurement PWV.

## Method

### Study population

Ten young, healthy volunteers participated in the study. They consisted of five men and five women between the age of 20-30 years old. They were recruited from hospital staff, family members and friends. To generate a healthy population, people with known cardiovascular disease and diabetes were not included. Another exclusion criteria was known hypertension (defined as systolic blood pressure ≥140 mmHg or diastolic blood pressure ≥90 mmHg) as this directly influences the PWV (5).

### Pulse wave velocity

The PWV were simultaneously measured with bioimpedance and tonometry in the *a. femoral, a. popliteal, a. dorsalis pedis* and *a. posterior tibial* in one leg.

### Bioimpedance

**Figure 1:**
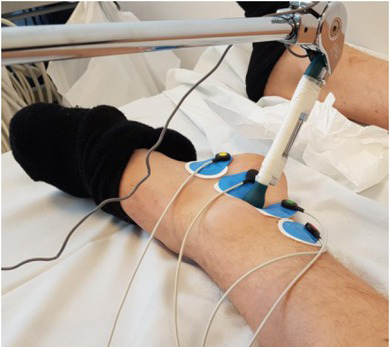
Illustration of posterior tibial artery with the tonometer between the four electrodes.

First the artery was located by palpation, before four Ambu® Blue Sensor electrodes were attached to measure bioimpedance through the skin (figure 1). Before placing the electrodes, the skin was washed with alcohol to increase the electrical contact of the electrodes. This could improve the electrical contact by removing the sebum from the skin surface (14). The two outer electrodes provided current and the two inner electrodes measured the differential voltage. Between the two inner electrodes the pulsation was felt best. It had a distance of two centimetres, as this is a good separation distance to detect the pulse wave and enough space to place the tonometer (15). All four electrodes were connected to the IPG bioimpedance instrument (MFIA Impedance Analyzer Precision LCR Meter 500 kHz / 5 MHz, Zurich Instruments) with the software LabOne. The settings used in MFIA for the frequency was 50 kHz, the sampling rate was 838 Hz and excitation voltage was 300mV. Additionally, a battery was used for the MFIA for electrical safety.

For the data analysis, the negative value of the modulus was used, because this was the most straightforward parameter and the most commonly used parameter in impedance cardiography.

### Tonometer and ECG

The SphygmoCor provided both pulse waveforms from the selected artery and an ECG. The R-peak from the ECG is a widely used reference point of the pulse wave starting from the heart (16).

The tonometer was placed in the middle of the four electrodes and was held by a stable holder so it could not be moved during the measurements, see figure 1. The SphygmoCor signals were sampled by the IPG bioimpedance instrument, so the pulse waveforms and ECG were both simultaneously recorded on the LabOne software, also with a sampling rate of 838 Hz.

### Data analysis

Each dataset was analysed and plotted with Matlab. To estimate the PWV the pulse arrival time (PAT) and the distance between the heart and the target point were measured. Fiducial time points are used to obtain PAT. The fiducial time point used for the tonometer was obtained with the tangent intersection method, which is one of the most effective methods for pressure pulse waves. The tangent intersection method is defined as the intersection in the pulse cycle of the horizontal line of the minimum point and the tangent of the initial systolic upstroke (17). The optimal fiducial time point had yet to be determined for bioimpedance. The following methods were tested for bioimpedance: the tangent intersection method, the peaks of the waves method and the maximum slope method. The fiducial time points and the R peak from the ECG were marked using Matlab. Their time difference was calculated and considered as the PAT. The distributions of the PAT outcomes for each fiducial time point method were compared with the results of the tonometer. The method that had the most similar outcomes as the tonometer was used. Subsequently the median and mode of the PAT over all heartbeats for each artery from both the tonometer and the bioimpedance were determined and compared between the devices. The value that was most similar between the devices was used for calculating the PWV.

The distance was measured with a measuring tape. The distance was measured from the heart to the umbilicus to the *femoral artery* to the other arteries. Subsequently the distance was divided by the PAT which provides the PWV.

To determine the precision of bioimpedance-based PAT from beat to beat, the interquartile ranges for every PAT from every artery from all the participants were determined for both devices. Subsequently a comparison of all the interquartile ranges for every artery and device was made. The average interquartile range of the two devices were calculated and compared with each other.

### Protocol

To minimalize the influence of blood pressure on PWV the following actions were taken: participants were asked not to eat, drink caffeine, smoke or use snus three hours and no alcohol use ten hours before the measurements; they had to lie down in a supine position for at least ten minutes before the measurement started; during the measurements participants were encouraged not to speak or sleep and the room was set on room temperature (between 22-24 °C)(5,18).

During resting three 3M electrodes were placed on the chest for the ECG. After ten minutes the blood pressure was measured by the auscultatory method according to Korotkoff in both arms. The arm with the highest blood pressure is measured one more time. The mean blood pressure is calculated from that arm and is considered as the blood pressure from the participant.

Subsequently the PWV was measured for five minutes in the *a. dorsalis pedis, a. posterior tibial, a. femoral* and *a. popliteal* according to the description mentioned earlier. The posterior artery is done last so the moving of the participant is minimalised as the participant is asked to turn around to lie on his stomach during this measurement.

After those five minutes, the distance between the heart and the four measured points of the arteries were measured.

### Informed consent

Informed consent has been obtained from all individuals included in this study.

### Ethical approval

The research has been complied with all relevant national regulations, institutional policies and in accordance with the tenets of the Helsinki Declaration. All data are anonymized.

## Results

### Study population

Ten subjects were included in the study. In one subject the quality of both the tonometer and bioimpedance did not have sufficient quality for precise analysis and this subject was therefore excluded, leaving nine test subjects for final analysis (table 1). In addition, measurements from one *a. dorsalis pedis*, one *a. femoralis* and one *a. popliteal* were removed from the data due to poor signal quality hampering pulse detection from either tonometer or bioimpedance signals.

**Table 1:** Main characteristics of the participants. Values are numbers or median [Q1-Q3]. BMI indicates the body mass index (weight/height^2^).

Variables	Value
Test subjects, *n* (males/females)	9 (5/4)
Age, years	24 [22-24]
Height, cm	184 [175-186]
Weight, kg	73.6 [64.6-78.6]
BMI, kg/m^2^	21.7 [20.3-22.7]
Systolic blood pressure, mmHg	108 [99-114]
Diastolic blood pressure, mmHg	69 [68-71]
Heart rate, beats/minute	59 [52-64]

### Method of analysis

**Figure 2:**
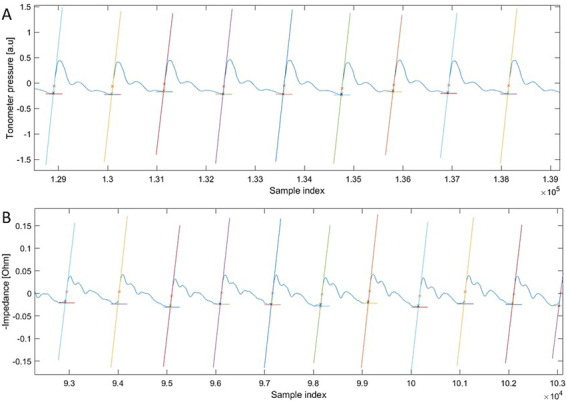
Analysis with the intersecting tangents method of the pulse wave forms to estimate the pulse arrival time showing part of two example recordings. (A) Tonometer. (B) Negative value of bioimpedance.

The different methods to determine the fiducial time points for bioimpedance were first compared. From these methods the intersecting tangent method seemed to be most suitable. It is the same method used in tonometer and the results were most similar to the results of tonometer (17). The intersecting tangent method for tonometer and bioimpedance is shown in figure 2.

### Relation between bioimpedance and tonometer

**Figure 3:**
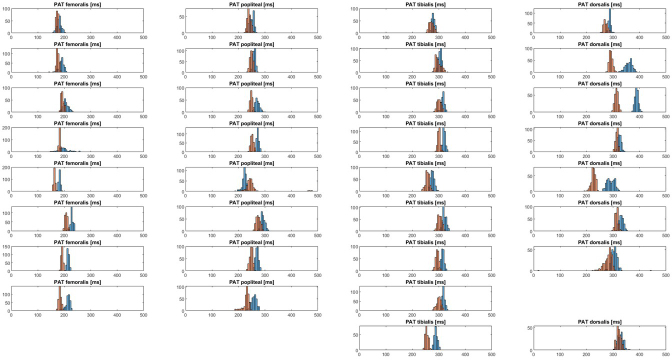
An indication of the difference between the tonometer (orange) and the bioimpedance (blue) for the estimated pulse arrival time (PAT) in milliseconds displayed in a distribution. The PAT was estimated in the a. femoralis, a. popliteal, a. tibialis posterior and a. dorsalis pedis in nine healthy participants during a five minute measurement. Each column represents an artery, and each row represents a participant. The a. dorsalis from one subject and a. femoralis and a. popliteal from another were removed from the data due to poor signal quality hampering pulse detection from either tonometer or bioimpedance signals.

**Figure 4:**
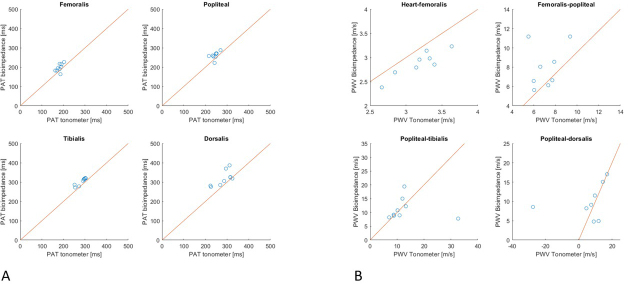
The relation between bioimpedance and tonometer measurements plotted along an x=y line in the a. femoralis, a. popliteal, a. tibialis posterior and a. dorsalis pedis using the mode from the nine healthy participants for (A) the estimated pulse arrival time (PAT) in milliseconds and (B) the calculated pulse wave velocity (PWV) in meters per second based on the PAT modes and measured distances between the arteries.

Figure 3 shows the estimated PAT from every measured pulse wave obtained by bioimpedance and tonometer. Each subplot shows a histogram of the results of five minutes measurement. The mode of the PAT was determined for every individual artery for each device. To compare bioimpedance with tonometer they are displayed with a x=y line in figure 4. Figure 4A shows the relation for the PAT. Figure 4B shows the relation for the calculated PWV based on the PAT modes and the measured distances.

**Figure 5:**
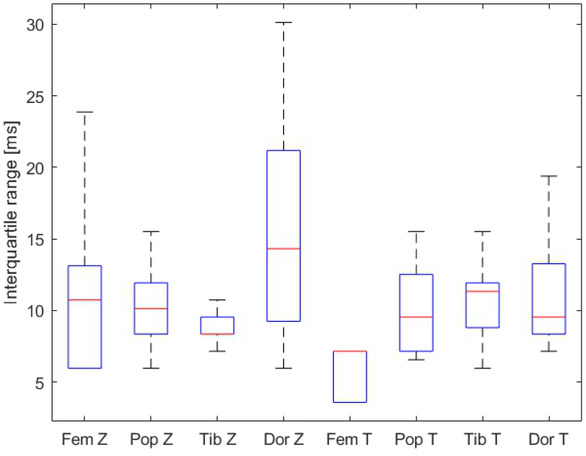
The distribution is displayed in boxplots, which are made of the interquartile ranges of the pulse arrival times (PAT) in milliseconds (ms) estimated by the bioimpedance (Z) and tonometer (T) from the nine healthy participants for the a. femoralis, a. popliteal, a. tibialis posterior and a. dorsalis pedis. First the interquartile ranges of the PAT for every artery from all the participants were determined. Subsequently a distribution of these interquartile ranges was made. The average interquartile range for bioimpedance (Z) is 11.48 ms and for tonometer (T) is 9.44 ms in all arteries.

All interquartile ranges of the PAT for every artery from all the participants were determined. Subsequently a distribution of these interquartile ranges was made. Figure 5 shows that distribution of the interquartile ranges of the PAT-modes per artery as boxplots. The average interquartile range for the bioimpedance is 11.48 ms and for the tonometer 9.44 ms.

**Figure 6:**
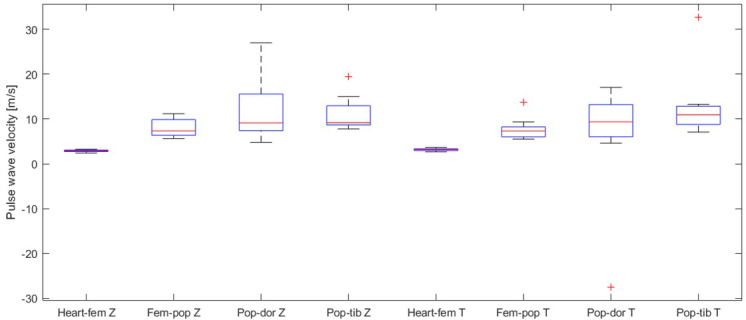
The distribution of the estimated pulse wave velocity (PWV) in meters per second (m/s) between the heart-a. femoralis; a. femoralis-a. popliteal; a. popliteal-a. tibialis posterior and a. popliteal-a. dorsalis from nine young healthy subjects by bioimpedance (Z) and tonometer (T) based on the mode of the pulse arrival time (PAT) in the arteries and distance between the measured sides.

Figure 6 shows a distribution of the estimated PWV over all participants based on the PAT modes from the arteries. Median values of the PWV estimated by bioimpedance were as follows: heart-femoral segment 2.90 m/s; femoralpopliteal 7.34 m/s; popliteal-dorsalis pedis segment 9.12 m/s and popliteal-tibialis posterior segment 9.18 m/s. Median values of the PWV by the tonometer were as follows: heart-femoral segment 3.19 m/s; femoral-popliteal segment 7.33 m/s; popliteal-dorsalis pedis segment 9.36 m/s and popliteal-tibialis posterior segment 10.94 m/s.

The mean and the confidence interval for the bioimpedance is as follows: heart-femoral segment 2.88 (2.69, 3.06) m/s; femoral-popliteal segment 7.99 (6.48, 9.51) m/s; popliteal-dorsalis pedis segment 11.81 (7.21, 16.41) m/s and popliteal-tibialis posterior segment 11.15 (8.66, 13.67) m/s. The results for the tonometer were the following: heart-femoral segment 3.17 (2.97, 3.36) m/s; femoralis-popliteal segment 7.79 (6.14; 9.44) m/s; poplitealdorsalis pedis 5.84 (-3.89, 15.58) m/s and popliteal-tibialis posterior 12.94 (7.92, 17.95) m/s.

**Figure 7:**
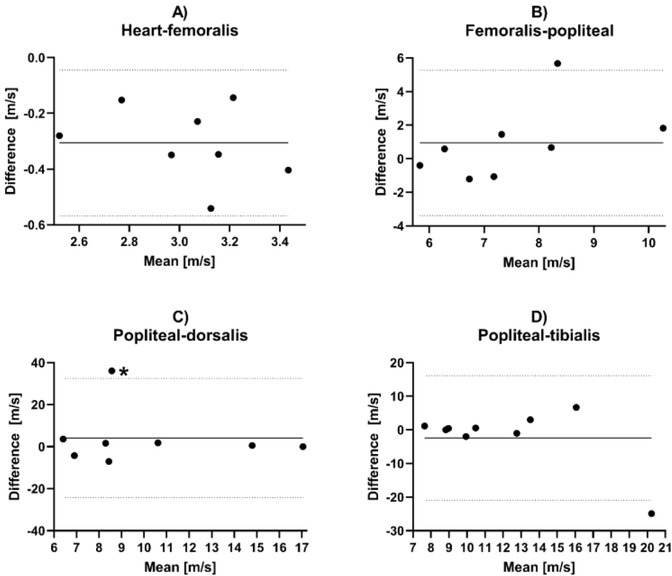
Bland-Altman plots for pulse wave velocity estimates for IPG method versus tonometry, showing the difference between the paired IPG and tonometer estimates versus the paired means. The bias is shown in the solid line and the 95% limits of agreement in dashed lines. *This pair had a negative mean value due to a large negative value for the tonometer estimate. In this case, the IPG estimate only was used as the mean value.

For further graphical comparison between the methods, Bland-Altman plots for the estimated PWV of all segments are provided in figure 7.

## Discussion

The aim of this study was to investigate if bioimpedance can be a suitable technique to measure the pulse wave velocity (PWV) in the legs as an alternative for the tonometer. We estimated with both methods, PWV in the a. fermoralis, a. popliteal, a. tibialis posterior and a. dorsalis pedis in nine young healthy participants. The bioimpedance and the tonometer obtained the pulse waves from the measured arteries. Gated by the R-wave in the ECG signal, the pulse arrival time (PAT) was estimated using a self-written script in Matlab. The PWV was estimated by dividing the distance from the heart to the femoral artery and between the measured arteries by the PAT. The results indicate that IPG may be a suitable technique to measure PWV. The estimated PWV results from bioimpedance and from the tonometer seem fairly in line with each other, see figure 6. The results of individual subjects seem to be in agreement with each other as well when they are illustrated in a scatter plot where both devices are compared with an x=y line (figure 4). These results support the hypothesis of the present study.

Koivistoinen *et al*. (19) used whole-body impedance cardiography in the aortic arch and the popliteal artery in a healthy population (age 25-41 years). They reported the PWV in male and female was 7.7 ± 1.4 m/s and 7.2 ± 1.2 m/s respectively (18). Rossow *et al*. (20) measured the PWV in women with the SphygmoCor in the carotid-femoral and femoral-tibialis posterior segments. The PWV in the younger group (age 19-25 years (22 ± 2)) was in the carotid-femoral 5.8 m/s and in the femoral-tibialis posterior arteries 8.5 m/s (19). Hashimoto *et al*. (21) used the tonometer of the SphygmoCor to measure the PWV in a healthy group (mean age 55±14 years) and measured a PWV of 7.8 (6.8-9.3) m/s in the carotid-femoral arteries and in the femoral-dorsalis pedis a PWV of 9.2±1.5 m/s (21).

The results of the present study are mostly in agreement with the previous studies, except for the estimated PWV between the heart and the femoral artery. We measured the following median PWV with bioimpedance: heart-femoral segment 2.90 m/s; femoral-popliteal segment 7.34 m/s; popliteal-dorsalis pedis segment 9.12 m/s and poplitealtibialis posterior segment 9.18 m/s. For the tonometer the median estimated PWV was in the heart-femoral segment 3.19 m/s; femoral-popliteal segment 7.33 m/s; poplitealdorsalis pedis segment 9.36 m/s and popliteal-tibialis posterior segment 10.94 m/s. A possible explanation for this difference could be that the ECG was not correctly synchronised with the other devices, as this is the only ECG dependent segment. The other PWV segments were calculated based on the differences in PAT, cancelling out possible signal lag of the ECG sampling, provided that it is constant.

Just as seen in the previous studies, it should be noted that the PWV increases when the artery is more distally. Two reasons for this are the change in diameter and change in wall properties from the aorta to the peripheral arteries. When the distance from the heart increases, the amount of elastin decreases, and the amount of collagen increases in the vessel walls. Elastin causes the ability for an artery to stretch, while collagen causes stiffening of the vessel wall (4,21).

A remarkable result is that it seems that the tonometer estimates a slightly higher PWV than the bioimpedance (figure 3 and 4). However, when the mean and the confidence intervals from the estimated PWV are compared, it seems that there was no statistical difference detectable, probably due to the given sample size of the study.

Another interesting result is that it seems that the bioimpedance signal has a third wave which is not present in the tonometer signal (figure 2). A possible explanation for this is that the bioimpedance is more sensitive to various changes in the blood vessel compared to the tonometer. The bioimpedance is affected by changes in blood volume, blood velocity and blood composition (haematocrit, the ratio of volume of red blood cells to the total volume of the blood), while the tonometer is only dependent on the pressure difference (23,24). The signal of the tonometer shows two peaks, the first peak is a result from the ejection of the left ventricle and the second peak from the wave reflections from the peripheral arteries (25). The third peak in the bioimpedance signal could be the dicrotic wave, which is a result from the dicrotic notch. The dicrotic notch reflects the aortic valve closure, which is partly closed by the reflection wave. It represents the end of the systole and the beginning of the diastole (26). The dicrotic notch affects the pulse pressure and the blood flow (27,28). As the bioimpedance detects both change in volume and blood flow, it could be more sensitive for the dicrotic notch than the tonometer. The dicrotic wave could be of clinical relevance as it represents etiologic factors. For example aortic valve diseases or an infectious that causes arterial vasodilatation and a compliant aorta and arterial tree (29,30).

Another difference between the devices that should be noted is the ease of use of the bioimpedance compared to the tonometer. During the measurements we observed that finding the bioimpedance signal was easier than finding the signal of the tonometer. Moreover, when a participant moved the bioimpedance signal recovered, while often the signal of the tonometer had to be found again.

There are limitations of this study that should be considered. Although the analogue ECG and tonometer signals were streamed directly from the Sphygmocor device with a high sampling rate (838 Hz) into the Zurich Instruments device (with a <1 ms delay between channels), the devices were not synchronised using trigger signals before the measurements. There could be a delay in the ECG compared to the SphygmoCor and/or IPG that caused the low PWV between the heart and the femoral artery and probably incorrect results. However, we believe that this possible error would only affect the heart to femoral estimates (as the other estimates use differences in pulse arrival times) assuming that such device lag would be constant. The heart to femoral PWV is also the least relevant segment in this context of evaluating peripheral pulse wave velocities. Another limitation that could influence the results is the method of measuring distances between the measured sides. The measured distance is not the real distance of the artery tracts, as it is measured on the skin. A third limitation is the number of participants. Only nine volunteers participated in this pilot study, which results in a low statistical power.

Despite these limitations, our results do provide an indication for the use of bioimpedance for estimating PWV as the estimated PWVs seem valid because they are in line with each other and with other studies. Furthermore, the estimated PAT was reliable as there was a small variation between the beats. These variations were also comparable between the two methods (figure 5). However, more research will be necessary to validate this technique. Future studies should focus on a larger sample, using devices that are synchronised with each other and using simultaneous methods that use dual impedance measurement and pulse transit times for PWV measurement (without the need for an ECG).

## Conclusion

Although further research is needed, the present study indicates that the use of IPG may be good alternative for estimating PWV in the legs and might have a potential to be implemented for clinical use in the future.
